# Associations of noninvasive measures of adiposity with aortic stiffness and microvascular function: The Jackson Heart Study

**DOI:** 10.1016/j.ajpc.2026.101449

**Published:** 2026-01-24

**Authors:** Carey R. Williams, Brielle Quarles, Alain G. Bertoni, Paul Muntner, Emelia J. Benjamin, Ramachandran S. Vasan, Gary F. Mitchell, Ervin R. Fox, Leroy L. Cooper

**Affiliations:** a Department of Biology, Tougaloo College, Tougaloo, MS, United States; b Warren Alpert Medical School of Brown University, Providence, RI, United States; c Biology Department, Vassar College, 124 Raymond Ave., Box 70, Poughkeepsie, NY 12604, United States; d Yale School of Nursing, New Haven, CT, United States; e Department of Epidemiology and Prevention, Division of Public Health Sciences, Wake Forest School of Medicine, Winston-Salem, NC, United States; f Perisphere Real World Evidence, LLC, Austin, TX, United States; g Boston University and NHLBI’s Framingham Study, Framingham, MA, United States; h Evans Department of Medicine, Boston Medical Center, Boston, MA, United States; i Whitaker Cardiovascular Institute, Boston University Chobanian and Avedisian School of Medicine, Boston, MA, United States; j Cardiology and Preventive Medicine Sections, Department of Medicine, Boston University Chobanian and Avedisian School of Medicine, Boston, MA, United States; k Department of Epidemiology, Boston University School of Public Health, Boston, MA, United States; l The University of Texas School of Public Health San Antonio, San Antonio, TX, United States; m The University of Texas Health Science Center, San Antonio, TX, United States; n Cardiovascular Engineering, Inc., Needham, MA, United States; o Division of Cardiovascular Diseases, Department of Medicine, University of Mississippi Medical Center, Jackson, MS, United States

**Keywords:** Hemodynamics, Epidemiology, Vascular biology, Obesity, Aortic stiffness

## Abstract

**Introduction:**

Studies suggest that vascular function measures may be more strongly associated with body fat distribution than global adiposity indices.

**Methods:**

In Jackson Heart Study participants at examination 2 (2007-2009), we assessed anthropometric obesity indices (body mass index and waist-to-height ratio) and visceral and subcutaneous adipose tissue volume via multidetector computed tomography (CT) scanning. Subsequently, a subset of participants underwent ancillary evaluation of arterial hemodynamics (2012-2017) to assess measures of aortic stiffness (carotid-femoral pulse wave velocity [CFPWV], forward wave amplitude, and characteristic impedance) and microvascular function (brachial arterial flow velocity before and after reactive hyperemia). We used multivariable linear regression models, adjusted for cardiovascular disease risk factors, to relate adiposity measures with arterial hemodynamic measures.

**Results:**

In 1276 participants (mean age 63 years, 66% women), higher body mass index was associated with lower negative inverse CFPWV (niCFPWV, estimated *β* per SD=-0.8±0.02; *P*=0.001) and characteristic impedance (*β*=-0.08±0.03; *P*=0.004). However, higher visceral adipose volume was associated with higher niCFPWV (*β*=0.14±0.03; *P*<0.001) and forward wave amplitude (*β*=0.08±0.03; *P*=0.002) in multivariable models that further adjusted for body mass index. Additionally, higher waist-to-height ratio (*β*=0.09±0.03; *P*=0.001) and body mass index (*β*=0.10±0.03; *P*<0.001), but not visceral or subcutaneous adiposity, were related to higher baseline brachial flow velocity. Adiposity measures were not related to hyperemic brachial flow velocity.

**Conclusion:**

Regional and global adiposity measures may have differing associations with aortic stiffness and microvascular function. Visceral fat may represent a more specific and pathophysiologically-relevant fat depot with direct links to vascular dysfunction. Assessing fat distribution, not just overall adiposity, may be more important when evaluating cardiovascular risk.

## Graphical abstract

**Figure:**
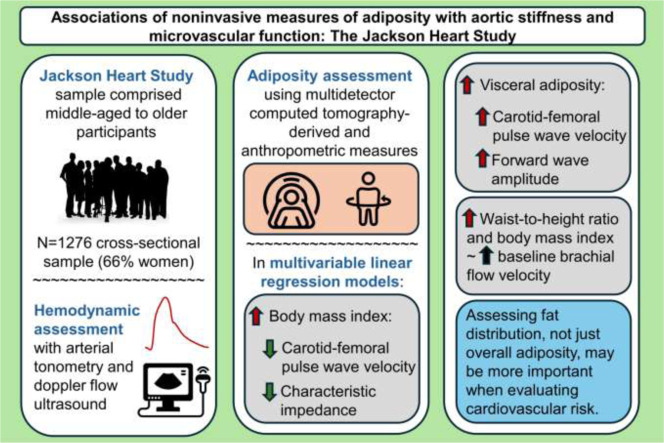

## 1 Introduction

The prevalence of obesity has been increasing in the United States and globally; therefore, obesity-associated co-morbidities are an increasing public health concern [1]. African-American adults have a higher prevalence of obesity (body mass index ≥30) compared with non-Hispanic White adults, and the disparity in obesity is exaggerated in women and begins to manifest early in life [2,3]. With high mortality rates attributable to cardiovascular disease (CVD), African-American adults have a disproportionate CVD burden compared to other racial and ethnic groups in the United States [4]. Although multiple studies have reported that high adiposity is strongly related with CVD risk factors and events [[5], [6], [7]], the mechanisms that contribute to higher CVD risk have not been completely elucidated.

Markers of aortic stiffness [8,9] and downstream microvascular dysfunction [10] are strong risk factors for CVD events. Recent studies suggest that arterial stiffening occurs earlier in life and accelerates faster in Black individuals [11,12], which may make them more susceptible to CVD. Additionally, studies examining relations between aortic stiffness and obesity in adults [[13], [14], [15], [16]] have suggested that body fat partitioning compared to anthropometric obesity indices may be more strongly correlated to aortic stiffness measures. Differences in associations among various adiposity measures with microvascular function are less studied. However, assessment of fat distribution as compared with overall global adiposity may be more informative when evaluating overall cardiovascular risk. We hypothesize that higher adiposity is associated with elevated aortic stiffness and worse microvascular function and that the strength of association may vary by index of adiposity. Additionally, we hypothesize that associations of adiposity measures with vascular measures are modified by sex and age. Thus, we aim to assess the relations of computed tomography (CT)-derived and anthropometric measures of adiposity with measures of aortic stiffness and microvascular function in a sample of Jackson Heart Study (JHS) participants.

## 2 Methods

Our study followed the Strengthening the Reporting of Observational Studies in Epidemiology (STROBE) reporting guidelines [17]. The procedure for requesting data from the JHS can be found at https://www.jacksonheartstudy.org/.

### 2.1 Participants

JHS is a community-based cohort study investigating risk factors for CVD in Black individuals; the details and design of the JHS have been described [18,19]. JHS participants were recruited in 2000 to 2004 from 3 counties surrounding Jackson, MS (Hinds, Madison, Rankin). For the current analysis, a subset of participants (N=2882) underwent multidetector CT scanning as part of the second JHS Examination, which occurred in 2007-2010; one participant had incomplete CT assessment and was excluded. Of the 2881 participants with complete CT assessment, 2071 attended an ancillary visit overlapping with the third examination cycle (2009-2013), with the arterial tonometry assessment of central and microvascular hemodynamics conducted (2012-2017). Written informed consent was obtained from all study participants at each study visit, and the study was approved by the institutional review boards of University of Mississippi Medical Center, Jackson State University, and Tougaloo College.

### 2.2 Assessment of aortic stiffness

Aortic stiffness was assessed using applanation tonometry with participants in the supine position after a 5-minute rest [9]. Using a custom tonometer, we obtained arterial tonometry with simultaneous electrocardiography from brachial, radial, femoral, and carotid arteries. We obtained 2-dimensional echocardiographic images of the left ventricular outflow tract along the parasternal long axis followed by pulsed Doppler of the left ventricular outflow tract. At the time of primary acquisition, we digitized and then transferred the data to a core laboratory (Cardiovascular Engineering, Inc., Needham, MA) where technicians performed analyses blinded to clinical data. Using the electrocardiographic R-wave as a reference, we signal-averaged tonometry waveforms [9]. We used systolic and diastolic blood pressures (obtained during tonometry) to calibrate the signal-averaged brachial waveforms. We integrated the brachial waveform to calculate mean arterial pressure and used diastolic pressures and the integrated mean arterial pressure to calibrate carotid pressures [20]. We used the calibrated carotid pressure as a surrogate for central pressure [20]. For carotid-femoral transit distance, we adjusted for parallel transmission as previously described [21]. We calculated carotid-femoral pulse wave velocity (CFPWV) as the ratio of the adjusted transit distance and the pulse transit time difference between carotid and femoral site. We calculated central pulse pressure as the difference between carotid systolic and diastolic blood pressures. Calibrated carotid pressure and aortic flow waveforms were used to compute characteristic impedance and to perform wave separation analysis [9]. We defined forward pressure wave amplitude (FWA) as the difference between pressure at the foot and at the peak of the forward pressure waveform by performing time domain wave separation analysis using central pressure and flow [21].

### 2.3 Assessment of microvascular function

Microvascular function was assessed using ultrasound image acquisition and analyses as described previously [22,23]. We acquired brachial artery Doppler flow at baseline and following five minutes of ischemia that was produced by inflating a cuff positioned on the forearm. Technicians placed the cuff just distal to the antecubital fold and inflated to approximately 50 mm Hg above systolic blood pressure. After cuff deflation, sonographers monitored and recorded flow (for 15 seconds after cuff release) until flow peaked. We assessed the brachial artery images and Doppler flow with a Siemens Acuson S2000 ultrasound system mounted with 4Vc and 9L4 transducers using a carrier frequency of 4.0 MHz and an insonation angle of approximately 60°, and we digitized ultrasound data during acquisition and transferred those data to the core laboratory (Cardiovascular Engineering, Inc., Needham, MA) for blinded analyses. Using a semi-automated signal-averaging technique [24]. we analyzed flows from the digitized Doppler audio data and visually confirmed timing of peak flow from a raw spectral analysis of distinct beats. We labeled 3 to 5 beats (representing the peak flow) for inclusion in the signal-averaged spectrum. Using the ECG as a fiducial point, we signal-averaged flow spectra and corrected them for actual insonation angle.

### 2.4 Adiposity assessment using multidetector CT scanning

Visceral adipose tissue (VAT) and subcutaneous adipose tissue (SAT) were used as quantitative volumetric measures of adipose tissue deposits as previously described [25,26]. Using a 16-channel multidetector CT, images from two slices preceding the center of the L4–L5 disk space and 12 slices following it were acquired. The abdominal muscular wall contours were initially outlined manually. Fat volumes within various compartments were then quantified using a semiautomatic segmentation approach. Volume analysis was performed with the Advantage Windows software (GE Healthcare, Waukesha, WI), which classified each voxel based on a tissue attenuation range for fat between −190 and −30 Hounsfield units. The total volumes of VAT and SAT were calculated by summing the respective voxels across 24 slices and reported in cubic centimeters.

### 2.5 Clinical evaluation and definitions

We assessed age, sex, smoking status (current smoking vs. nonsmoking), and use of antihypertensive medications and lipid-lowering medication via questionnaire. Heart rate and mean arterial pressure were assessed during tonometry. Prevalent CVD was defined as history of myocardial infarction, coronary heart disease, heart failure, and stroke as previously described [27]. We measured serum cholesterol levels from fasting blood tests. We calculated the ratio of total to high-density lipoprotein cholesterol. We defined presence of diabetes as fasting serum glucose ≥126 mg/dL, use of glucose-lowering medications within two weeks of the research center visit, or prior physician-diagnosed diabetes. To assess global adiposity, we measured height, weight, and waist circumference during the exam. We calculated BMI as the ratio of body weight in kilograms and the square of height in meters. The waist-to-height ratio was calculated by dividing waist circumference in meters by height in meters. Metabolic syndrome was defined as meeting ≥3 of 5 criteria: (1) high waist circumference (≥102 cm in men; ≥88 cm in women); (2) high fasting triglyceride (≥150 mg/dL/≥1.7mmol/L or treatment for elevated lipids); (3) high blood pressure (≥130 mm Hg systolic blood pressure, ≥85 mm Hg diastolic blood pressure, or treatment for hypertension); (4) low high-density lipoprotein cholesterol (<40 mg/dL in men; <50 mg/dL in women); and (5) high fasting glucose (≥100 mg/dL or treatment for elevated glucose) [28,29].

### 2.6 Statistical analyses

**Fig. 1:**
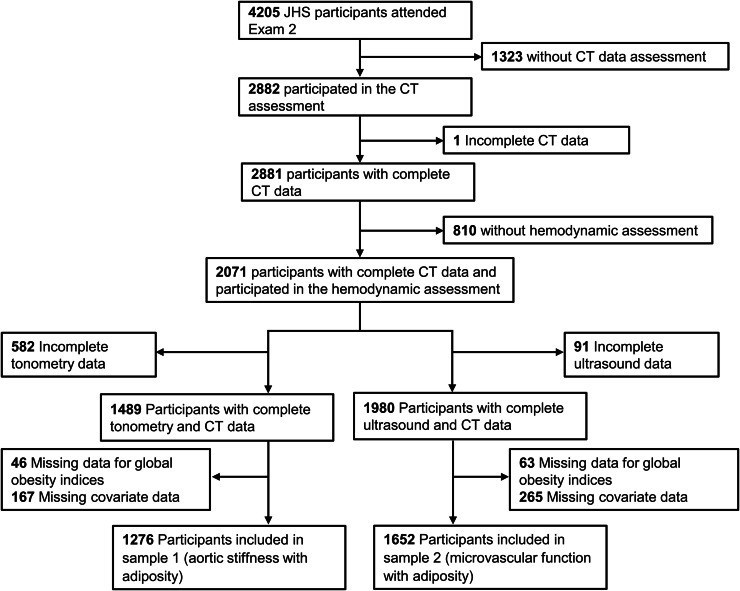
Flow chart for inclusion of participants for the present analyses.

The analysis was conducted using two samples: participants with complete data on multidetector CT and tonometry assessment of aortic stiffness (sample 1) and participants with complete data on multidetector CT and ultrasound assessment for microvascular function (sample 2). To assess relations of aortic stiffness with measures of adiposity (sample 1), we excluded participants for the following reasons: missing or incomplete tonometry data (N=582), global adiposity data (N=46), or covariate data (N=167). To assess the relations between measures of microvascular function and measures of adiposity (sample 2), we excluded participants for the following reasons: missing or incomplete ultrasound data (N=91), global adiposity data (N=63), or covariate data (N=265). Fig. 1 presents a flow chart of the samples for the current analysis. Sample characteristics for the included participants were tabulated. We calculated negative inverse CFPWV by inverting CFPWV to limit heteroscedasticity then multiplying by −1000 to convert units to ms/m and rectify directionality of associations with aortic stiffness. We transformed fasting blood glucose using natural logarithm to normalize its skewed distribution and standardized (mean=0, standard deviation=1) all continuous measures for modeling.

We assessed associations of adiposity measures with measures of aortic stiffness in sample 1 and measures of microvascular function in sample 2 using multivariable linear regression models. Based on review of the literature, we selected covariates *a priori* including age, age^2^, sex, heart rate, mean arterial pressure, diabetes, prevalent CVD, use of antihypertensive medication, use of lipid-lowering medication, fasting glucose, total/high-density lipoprotein cholesterol ratio, and smoking status. We further adjusted models for VAT and SAT for body mass index as well as the time between CT and tonometry or ultrasound assessment. As a sensitivity analysis, we repeated the primary analyses after excluding participants with prevalent CVD. In addition, we assessed the presence of effect modification by age (<63 and ≥63 years, the median age of the sample), sex, presence of diabetes, presence of metabolic syndrome, and extent of aortic stiffness for the microvascular models only by incorporating corresponding interaction terms into the analyses. We performed all analyses with SAS version 9.4 for Windows (SAS Institute, Cary, NC). Bonferroni-adjusted, 2-sided *P* values were used to assess statistical significance in each primary analysis, with adjustments based on an α level of 0.05. For interactions, we considered two-tailed *P*<0.05 statistically significant for the further assessment of relations using stratification.

## 3 Results

We present the sample characteristics of the 1276 JHS participants (mean age 63 years, 66% women) in Table 1. The sample comprised middle-age to older adults with a similar body mass index and a higher prevalence of women, diabetes, hypertension treatment, and smoking compared to the general adult population. However, the sample had a slightly lower prevalence of smoking and hyperlipidemia treatment.

**Table 1:** Clinical and demographic characteristics of the study sample (N=1276).

**Variable**	Value*
Age, years	63±10
Women, n (%)	837 (66)
Height, m	1.7±0.9
Heart rate, beats per minute	65±10
Mean arterial pressure, mm Hg	99±11
Total/high-density lipoprotein cholesterol ratio	3.6±1.1
Fasting glucose, mg/dL	97 (89, 108)
Medical History	
Antihypertensive medication, n (%)	943 (74)
Active smoking, n (%)	127 (10)
Lipid-lowering medication, n (%)	526 (41)
Prevalent cardiovascular disease, n (%)	93 (7)
Prevalent diabetes, n (%)	372 (29)
Adiposity Variables	
Waist-to-height ratio	0.60±0.08
Body mass index, kg/m^2^	31.0±5.9
Abdominal visceral adipose tissue, cm^3^	786±350
Abdominal subcutaneous adipose tissue, cm^3^	2249±971
Hemodynamic variables	
Carotid-femoral pulse wave velocity, m/s	11.0±4.2
Negative inverse carotid-femoral pulse wave velocity, ms/m	-101±31
Forward wave amplitude, mm Hg	54±16
Characteristic impedance, dyne∙s/cm^5^	270±111
Baseline flow velocity, cm/s (n=1652)	5.3±3.1
Hyperemic flow velocity, cm/s (n=1652)	46.8±18.4

All values are mean±standard deviation or median (25th, 75th percentile) except as noted.

In multivariable models, higher body mass index was associated with lower negative inverse CFPWV (estimated *β* per SD =-0.8±0.02; *P*=0.001) and characteristic impedance (*β*=-0.08±0.03; *P*=0.004) but not forward wave amplitude (Table 2). In models that further adjusted for body mass index, higher visceral adipose volume was associated with higher negative inverse CFPWV (*β*=0.14±0.03; *P*<0.001) and forward wave amplitude (*β*=0.08±0.03; *P*=0.002) but not characteristic impedance. Waist-to-hip ratio and subcutaneous adipose volume were not associated with any measure of aortic stiffness. Additionally, in multivariable models, higher waist-to-height ratio (*β*=0.09±0.03; *P*=0.001) and body mass index (*β*=0.10±0.03; *P*<0.001), but not visceral or subcutaneous adiposity, were related to higher baseline brachial flow velocity (Table 3); adiposity measures were not related to hyperemic brachial flow velocity. Sensitivity analyses excluding participants with prevalent CVD yielded results that were generally consistent with the primary analyses (**Supplemental Tables 1 and 2**).

**Table 2:** Associations of adiposity measures (per standard deviation change) with measures of aortic stiffness (N=1276).

Adiposity variable	niCFPWV	FWA	Z_C_
	*β*±SE (*P*)	*β*±SE (*P*)	*β*±SE (*P*)
Waist-to-height ratio	-0.03±0.02 (0.17)	-0.01±0.02 (0.61)	-0.07±0.03 (0.01)
Body mass index	-0.08±0.02 (0.001)	-0.01±0.02 (0.61)	-0.08±0.03 (0.004)
VAT*	0.14±0.03 (<0.001)	0.08±0.03 (0.002)	0.06±0.03 (0.05)
SAT*	-0.06±0.05 (0.17)	0.01±0.05 (0.79)	0.06±0.05 (0.26)

niCFPWV, negative inverse carotid-femoral pulse wave velocity. FWA, forward wave amplitude. Z_c_, characteristic impedance. VAT, abdominal visceral adipose tissue. SAT, abdominal subcutaneous adipose tissue. Regression estimates (*β*) followed by the standard error (SE) and *P* values. All coefficients represent SD difference in adiposity variables per SD difference in hemodynamic variables. All models are additionally adjusted for age, age^2^, sex, heart rate, mean arterial pressure, diabetes, prevalent cardiovascular disease, use of antihypertensive medication, use of lipid-lowering medication, fasting glucose, total/high-density lipoprotein cholesterol ratio, and smoking status.

*Models additionally adjusted for body mass index and time between computed tomography and tonometry assessment. Bonferroni-adjusted *P* values (*P*=0.05/12=0.0042) were used to assess significance of associations.

**Table 3:** Associations of adiposity measures (per standard deviation change) with measures of microvascular function (N=1652).

Adiposity variable	**Baseline flow velocity**	**Hyperemic flow velocity**
	*β*±SE (*P*)	*β*±SE (*P*)
**Waist-to-height ratio**	0.09±0.03 (0.001)	0.03±0.02 (0.21)
**Body mass index**	0.10±0.03 (<0.001)	0.04±0.02 (0.09)
VAT*	0.04±0.03 (0.19)	0.00±0.03 (0.96)
SAT*	-0.04±0.05 (0.47)	-0.04±0.05 (0.42)

Regression estimates (*β*) followed by the standard error (SE) and *P* values. All coefficients represent SD difference in adiposity variables per SD difference in flow velocity variables. All models are additionally adjusted for age, age^2^, sex, heart rate, mean arterial pressure, diabetes, prevalent cardiovascular disease, use of antihypertensive medication, use of lipid-lowering medication, fasting glucose, total/high-density lipoprotein cholesterol ratio, and smoking status.

*Models additionally adjusted for body mass index and time between computed tomography and ultrasound assessment. VAT, abdominal visceral adipose tissue. SAT, abdominal subcutaneous adipose tissue. Bonferroni-adjusted *P* values (*P*=0.05/8=0.006) were used to assess significance of associations

**Fig. 2:**
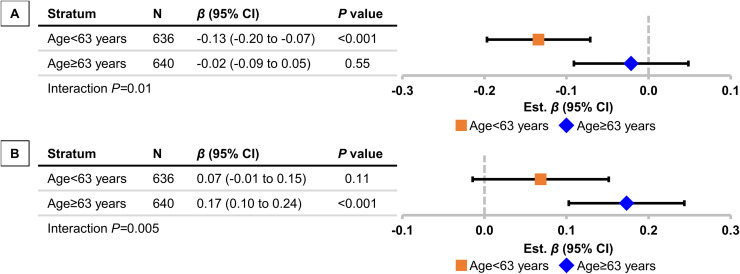
Effect modification by median age for associations of (A) body mass index (BMI) and (B) visceral adipose tissue (VAT) volume with negative inverse carotid-femoral pulse wave velocity (niCFPWV). Estimated effect sizes (*β*s), 95% confidence intervals, and *P* values are from linear regression models. All coefficients represent SD difference in adiposity variables per SD difference in niCFPWV. All models are additionally adjusted for age, age^2^**,** sex, heart rate, mean arterial pressure, diabetes, prevalent cardiovascular disease, use of antihypertensive medication, use of lipid-lowering medication, fasting glucose, total/high-density lipoprotein cholesterol ratio, and smoking status. The model in which VAT is the independent variable is further adjusted for BMI and time between computed tomography and tonometry assessment.

**Fig. 3:**
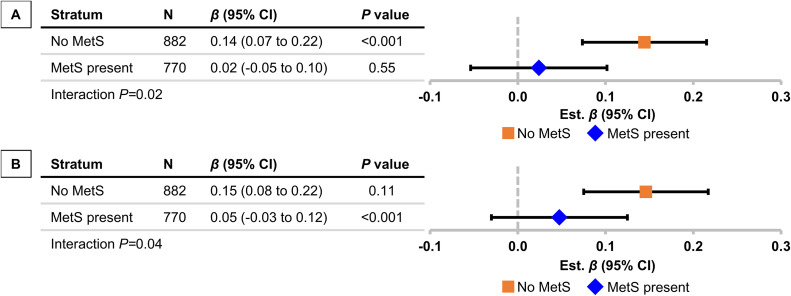
Effect modification by presence of metabolic syndrome for associations of (A) waist-to-height ratio and (B) body mass index with baseline brachial flow velocity. Estimated effect sizes (*β*s), 95% confidence intervals, and *P* values are from linear regression models. All coefficients represent SD difference in adiposity variables per SD difference in baseline brachial flow velocity. All models are additionally adjusted for age, age^2^**,** sex, heart rate, mean arterial pressure, diabetes, prevalent cardiovascular disease, use of antihypertensive medication, use of lipid-lowering medication, fasting glucose, total/high-density lipoprotein cholesterol ratio, and smoking status.

In the stratified analyses (Fig. 2; Supplemental Table 3), higher body mass index was associated with lower negative inverse CFPWV among younger but not older participants (*P*_interaction_=0.01), whereas higher visceral adipose volume was associated with higher negative inverse CFPWV among older but not younger participants (*P*_interaction_=0.005). Additionally, higher waist-to-height ratio and body mass index were associated with higher baseline flow velocity among participants without prevalent metabolic syndrome; this association was not significant among participants with prevalent metabolic syndrome (Fig. 3; Supplemental Table 4). Other interactions for the foregoing relations were not statistically significant (all *P-*values >0.05). There was no evidence of effect modification for the associations of higher visceral adipose volume with higher forward wave amplitude or higher body mass index with lower characteristic impedance.

## 4 Discussion

### 4.1 Principal findings

We observed that higher mean BMI was associated with lower mean aortic stiffness, particularly among younger participants. In contrast, higher VAT was associated with higher aortic stiffness, particularly among older participants. Waist-to-height ratio and SAT were not significantly associated with measures of aortic stiffness. Additionally, higher BMI and waist-to-height ratio were associated with higher baseline flow velocity, particularly among participants without metabolic syndrome. However, no measures of adiposity were associated with hyperemic flow velocity. Thus, these findings suggest regional and global adiposity measures may have differing associations with aortic stiffness measures. Additionally, higher global adiposity may contribute to higher peripheral arterial and microvascular basal tone.

### 4.2 Measures of adiposity and aortic stiffness

Consistent with prior work [14,30], higher VAT was associated with higher aortic stiffness in the JHS. Compared to subcutaneous fat, visceral fat is substantially more metabolically active and releases an array of pro-inflammatory cytokines and adipokines that lead to systemic and chronic inflammation, which can contribute to endothelial dysfunction and vascular stiffness [31]. Furthermore, visceral and subcutaneous fat have different profiles of adipokine secretion. Visceral fat produces more adipokines that promote vascular dysfunction, whereas, subcutaneous fat produces adipokine profiles that may be more vasoprotective (e.g., adiponectin, which has anti-inflammatory and vasodilatory properties) [32]. Excess visceral fat is associated with fat deposition in organs and the vasculature. For example, perivascular adipose tissue has been shown to exhibit a pro-inflammatory phenotype in obese individuals [33] and can secrete inflammatory cytokines that act locally and systemically to recruit other pro-inflammatory cells [34], further contributing to the formation of atherosclerotic lesions and endothelial dysfunction. Since inflammatory cytokines are known to upregulate several elastolytic enzymes, chronic inflammation may also disrupt the balance of elastin breakdown and synthesis [35]. Thus, pathologic vascular remodeling via inflammatory downregulation of arterial elastin and increased deposition of collagen can contribute to aortic stiffness.

Additionally, visceral fat is directly connected to the portal circulation and exposes the liver to free fatty acids and inflammatory mediators that may contribute to metabolic syndrome, which may have a bidirectional and reinforcing association with increased aortic stiffness [36]. Accumulation of visceral fat may increase intra-abdominal pressure [37], which can directly affect hemodynamics and increase the mechanical load on the aorta, contributing to stiffness [38]. We observed that among older participants, the relations for higher VAT with higher aortic stiffness was particularly strong. We posit that age-related aortic stiffening is intensified by the pro-inflammatory and metabolic effects of excess visceral fat. Alternatively, older participants have had longer exposure to metabolic and CVD risk factors that are strongly associated with visceral fat; therefore, this cumulative exposure may contribute to a stronger association of VAT with aortic stiffness among older individuals. Thus, the relation of higher VAT with higher aortic stiffness is likely multifaceted; more research investigating the metabolic, inflammatory, and mechanical mechanisms that underlie this association is warranted.

Discordant from CT measures of adiposity, global measures of adiposity generally were related to lower measures of aortic stiffness. This seeming paradox likely reflects potential confounding by body size and presence of lean mass as well as the differing biological roles of prevalent adipose tissue types. Since body mass index does not distinguish between fat and lean mass, participants with higher lean mass (e.g., men and younger participants) may have higher body mass index but healthier vascular profiles [39]. Furthermore, lean mass is associated with greater arterial distensibility due to higher cardiac output and shear stress, which may stimulate adaptive remodeling and help preserve elastin in the aorta [40]. This hypothesis is consistent with our observation that higher body mass index was associated with lower negative inverse CFPWV among younger but not older participants. Yet, we observed no association of global measures of adiposity with central hemodynamic measures, which suggests that these measures are more strongly associated with aortic geometry and wave propagation rather than with cardiac function (i.e., left ventricular ejection and incident wave generation). Alternatively, older individuals, who disproportionately have higher aortic stiffness, may have unintentional weight loss due to underlying morbidity or frailty (lowering body mass index). Higher body mass index can correspond to generally larger body size, which is associated with larger aortic diameter, which in turn is associated with lower aortic stiffness [41]. Global measures of adiposity do not distinguish fat from muscle, differentiate between subcutaneous and the more deleterious visceral fat, or account for regional fat distribution. As a result, individuals with similar body mass indices may have markedly different metabolic risk profiles depending on the distribution of adipose tissue. In contrast, CT-derived VAT provides a more precise and biologically relevant (though more resource-intensive) assessment of the adiposity most strongly implicated in aortic stiffening. However, further research is needed to clarify causal pathways linking specific fat depots to vascular function and to explore whether integrating advanced imaging into risk assessment improves risk prediction beyond conventional anthropometric measures.

### 4.3 Measures of adiposity and microvascular function

We observed significant associations of higher global adiposity measures with higher baseline flow velocity but not hyperemic flow velocity. Baseline brachial flow velocity is influenced by the density, tone, and structural characteristics of the forearm microvasculature [22,42], whereas hyperemic flow velocity reflects the near-maximal dilation of microvessels in response to ischemia-induced vasodilator production, such as nitric oxide [22,24,43,44]. Given that body mass index and waist-to-height ratio reflect general body size, our observations may indicate that larger persons have greater metabolic and perfusion demands at rest. For example, due to greater systemic tissue requirements, larger individuals have higher cardiac output and stroke volume, contributing to higher resting brachial blood flow velocities [45]. Therefore, the positive relation of global adiposity indices and higher baseline flow brachial flow velocity may be hemodynamic (reflecting overall circulatory demands) rather than reflecting local vascular function *per se*. Hyperemic brachial flow velocity reflects microvascular reactivity rather than hemodynamic load. We infer that adiposity is not a primary contributor to microvascular function in this sample. These findings contrast with prior studies in women, where obesity was associated with impaired microvascular function, specifically reduced postocclusive capillary recruitment [46]. The foregoing study assessed skin microvascular hyperemic response at the nailfold, whereas the current study assessed conduit artery hyperemia via brachial flow velocity. Therefore, differences in vascular level, measurement technique, or sample characteristics may account for the contrasting findings regarding the impact of adiposity measures on vascular function. Additionally, we acknowledge the possibility of residual confounding by lean mass. Since body mass index and waist-to-height ratio correlate with lean body mass and individuals with greater lean mass have higher muscle perfusion demands at rest [47], baseline brachial flow velocity may be higher in individuals with more muscle mass, without necessarily affecting reactive hyperemic flows. For example, in a sample of young, healthy men, Garten et al. observed that, unlike at rest, muscle mass did not determine the hyperemic flow response during exercise [47].

Global anthropometric measures of adiposity were related to baseline flow, while CT measures were not. Although visceral and subcutaneous fat depots reflect regional fat distribution, they may not directly influence resting brachial arterial flow. Instead, baseline flow is primarily governed by local microvascular characteristics (i.e., density, tone, and structure) with systemic factors like cardiac output and peripheral perfusion demands modifying flow magnitude in response to overall body size. Additionally, we adjusted relations of CT measures of adiposity with microvascular function with body mass index to account for body size, so the absence of significant associations suggests that regional fat depots may not independently affect microvascular function beyond the systemic effects of overall adiposity or body size.

### 4.4 Study limitations

The present study has limitations that should be considered. We employed a cross-sectional observational study design; this limits our ability to infer causal and temporal relations between arterial hemodynamic measures and measures of adiposity. The samples for this investigation were middle-aged to older Black participants from the Jackson, MS region; therefore, our findings may not be generalizable to younger individuals or individuals of other racial or ethnic groups or other regions. Since our analytic samples were drawn from two different examination cycles, our study is susceptible to survivorship bias. Although we adjusted models for known factors that contribute to aortic stiffness, the possibility of residual confounding by unknown and unmeasured factors remains. Consideration of these limitations should be balanced with acknowledgment of the study's strengths. The JHS is a well-characterized, community-based cohort designed to enhance understanding of vascular function in a society with increasing rates of obesity. Thus, in this study, we were able to investigate the relations of adiposity measures with measures of aortic stiffness and microvascular function in an underrepresented and understudied group using clinically-relevant vascular tonometry and ultrasound techniques.

## 5 Conclusion

In this cross-sectional study, regional and global adiposity measures demonstrated distinct associations with aortic stiffness measures. Additionally, higher global adiposity may contribute to higher peripheral arterial and microvascular basal tone. Therefore, we observed differing associations for regional and global adiposity measures with noninvasive measures of aortic stiffness and microvascular function. Our findings underscore the potential importance of assessing fat distribution, not just overall adiposity, when evaluating vascular risk and tailoring preventive strategies.

## Funding sources

Dr. Mitchell is funded by research grants HL094898, DK082447, HL107385, HL104184, and HL126136 from the National Institutes of Health. Dr. Benjamin was funded by research grants R01HL128914; 2R01 HL092577; American Heart Association 18SFRN34110082; 1R01HL141434; 2U54HL120163, and HHSN26820130047C. Dr. Cooper was funded by NHLBI grant K01HL161494.

## Disclosures

G.F.M. is owner of Cardiovascular Engineering, Inc., a company that designs and manufactures devices that measure vascular stiffness. The company uses these devices in clinical trials that evaluate the effects of diseases and interventions on vascular stiffness. G.F.M. also serves as a consultant to and receives grants and honoraria from Novartis, Merck, Bayer, Servier, Philips, and deCODE genetics and is an inventor on a pending patent application that discloses a method for estimating CFPWV and vascular age by using a convolutional neural network. The other authors report no conflicts.

## Ethical review statement

Written informed consent was obtained from all Jackson Heart Study participants at each study visit, and the study was approved by the institutional review boards of University of Mississippi Medical Center, Jackson State University, and Tougaloo College.

## JHS disclaimer

The views expressed in this manuscript are those of the authors and do not necessarily represent the views of the National Heart, Lung, and Blood Institute; the National Institutes of Health; or the U.S. Department of Health and Human Services.

## Author declaration

We wish to draw the attention of the Editor to the following facts which may be considered as potential conflicts of interest and to significant financial contributions to this work:

The authors wish to thank the staff and participants of the Jackson Heart Study. The Jackson Heart Study is supported by Contracts HHSN268201800010I, HHSN268201800011I, HHSN268201800012I, HHSN268201800013I, HHSN268201800014I, HHSN268201800015I from the National Heart, Lung, and Blood Institute (NHLBI) with additional support from the National Institute on Minority Health and Health Disparities (NIMHD). This manuscript has been reviewed by JHS for scientific content.

JHS disclaimer: The views expressed in this manuscript are those of the authors and do not necessarily represent the views of the National Heart, Lung, and Blood Institute; the National Institutes of Health; or the U.S. Department of Health and Human Services.

## Disclosures

G.F.M. is owner of Cardiovascular Engineering, Inc., a company that designs and manufactures devices that measure vascular stiffness. The company uses these devices in clinical trials that evaluate the effects of diseases and interventions on vascular stiffness. G.F.M. also serves as a consultant to and receives grants and honoraria from Novartis, Merck, Bayer, Servier, Philips, and deCODE genetics and is an inventor on a pending patent application that discloses a method for estimating CFPWV and vascular age by using a convolutional neural network. The other authors report no conflicts.

We confirm that the manuscript has been read and approved by all named authors and that there are no other persons who satisfied the criteria for authorship but are not listed. We further confirm that the order of authors listed in the manuscript has been approved by all of us.

We confirm that we have given due consideration to the protection of intellectual property associated with this work and that there are no impediments to publication, including the timing of publication, with respect to intellectual property. In so doing we confirm that we have followed the regulations of our institutions concerning intellectual property.

We further confirm that any aspect of the work covered in this manuscript that has involved human patients has been conducted with the ethical approval of all relevant bodies and that such approvals are acknowledged within the manuscript. Written informed consent was obtained from all Jackson Heart Study participants at each study visit, and the study was approved by the institutional review boards of University of Mississippi Medical Center, Jackson State University, and Tougaloo College.

We understand that the Corresponding Author is the sole contact for the Editorial process (including Editorial Manager and direct communications with the office). He is responsible for communicating with the other authors about progress, submissions of revisions and final approval of proofs. We confirm that we have provided a current, correct email address which is accessible by the Corresponding Author.

Signed by all authors as follows:

Carey R. Williams 9/15/2025

Brielle Quarles 9/15/2025

Alain G. Bertoni 9/15/2025

Paul Muntner 9/15/2025

Emelia J. Benjamin 9/15/2025

Ramachandran S. Vasan 9/15/2025

Gary F. Mitchell 9/15/2025

Ervin R. Fox 9/15/2025

Leroy L. Cooper 9/15/2025

## CRediT authorship contribution statement

**Carey R. Williams:** Writing – review & editing, Writing – original draft, Investigation, Formal analysis. **Brielle Quarles:** Writing – review & editing, Writing – original draft, Investigation. **Alain G. Bertoni:** Writing – review & editing, Conceptualization. **Paul Muntner:** Writing – review & editing, Conceptualization. **Emelia J. Benjamin:** Writing – review & editing, Funding acquisition. **Ramachandran S. Vasan:** Writing – review & editing. **Gary F. Mitchell:** Writing – review & editing, Supervision, Funding acquisition. **Ervin R. Fox:** Writing – review & editing, Supervision, Conceptualization. **Leroy L. Cooper:** Writing – review & editing, Writing – original draft, Visualization, Supervision, Project administration, Investigation, Funding acquisition, Formal analysis, Data curation.

## Declaration of competing interest

The authors declare the following financial interests/personal relationships which may be considered as potential competing interests:

Leroy L. Cooper reports financial support was provided by National Heart Lung and Blood Institute. Emelia J. Benjamin reports financial support was provided by American Heart Association Inc. Emelia J. Benjamin reports financial support was provided by National Heart Lung and Blood Institute. Gary F. Mitchell reports financial support was provided by National Heart Lung and Blood Institute. Gary F. Mitchell reports a relationship with Novartis, Merck, Bayer, Servier, Philips, and deCODE genetics that includes: consulting or advisory and funding grants. The Jackson Heart Study is supported by Contracts HHSN268201800010I, HHSN268201800011I, HHSN268201800012I, HHSN268201800013I, HHSN268201800014I, HHSN268201800015I from the National Heart, Lung, and Blood Institute (NHLBI) with additional support from the National Institute on Minority Health and Health Disparities (NIMHD). This manuscript has been reviewed by JHS for scientific content.

G.F.M. is owner of Cardiovascular Engineering, Inc., a company that designs and manufactures devices that measure vascular stiffness. The company uses these devices in clinical trials that evaluate the effects of diseases and interventions on vascular stiffness. G.F.M. is an inventor on a pending patent application that discloses a method for estimating CFPWV and vascular age by using a convolutional neural network. If there are other authors, they declare that they have no known competing financial interests or personal relationships that could have appeared to influence the work reported in this paper.

## Appendix Supplementary materials

Image, application 1
