# Supplementary material for: Associations of noninvasive measures of adiposity with aortic stiffness and microvascular function: The Jackson Heart Study
Source: Am J Prev Cardiol. 2026 Jan 24;26:101449. doi: 10.1016/j.ajpc.2026.101449 (PMC13084131; doi:10.1016/j.ajpc.2026.101449)
Supplement: Supplementary file 1 [file mmc1.pdf]

SUPPLEMENTAL MATERIAL

Associations of noninvasive measures of adiposity with aortic stiffness and microvascular function: The Jackson Heart Study

Authors' Names, Academic Degrees, and Affiliations: Carey R. Williams, BS,[a,b] Brielle Quarles, BA,[c,d] Alain G. Bertoni, MD, MPH,[e] Paul Muntner, PhD, MHS,[f] Emelia J. Benjamin, MD, ScM,[g,h,i,j,k] Ramachandran S. Vasan, MD,[g,l,m] Gary F. Mitchell, MD,[n] Ervin R. Fox, MD, MPH,[o] and Leroy L. Cooper, PhD, MPH[c]

[a]Department of Biology, Tougaloo College, Tougaloo, MS; [b]Warren Alpert Medical School of Brown University, Providence, RI; [c]Biology Department, Vassar College, Poughkeepsie, NY; [d]Yale School of Nursing, New Haven, CT; [e]Department of Epidemiology and Prevention, Division of Public Health Sciences, Wake Forest School of Medicine, Winston-Salem, NC; [f]Perisphere Real World Evidence, LLC. Austin, TX; [g]Boston University and NHLBI's Framingham Study, Framingham, MA; [h]Evans Department of Medicine, Boston Medical Center, Boston, MA; [i]Whitaker Cardiovascular Institute, Boston University Chobanian and Avedisian School of Medicine, Boston, MA; [j]Cardiology and Preventive Medicine Sections, Department of Medicine, Boston University Chobanian and Avedisian School of Medicine, Boston, MA; [k]Department of Epidemiology, Boston University School of Public Health, Boston, MA; [l]The University of Texas School of Public Health San Antonio, San Antonio, TX; [m]The University of Texas Health Science Center, San Antonio, TX; [n]Cardiovascular Engineering, Inc., Needham, MA; and [o]Division of Cardiovascular Diseases, Department of Medicine, University of Mississippi Medical Center, Jackson, MS

Supplemental Content:

Supplemental Table 1. Associations of adiposity measures (per standard deviation change) with measures of aortic stiffness in participants without cardiovascular disease (N=1183).

Supplemental Table 2. Associations of adiposity measures (per standard deviation change) with measures of microvascular function in participants without cardiovascular disease (N=1534).

Supplemental Table 3. Summary of interactions for relations of aortic stiffness with body mass index and abdominal visceral adipose tissue.

Supplemental Table 4. Summary of interactions for relations of baseline brachial flow velocity with waist-to-height ratio and body mass index.

Supplemental Table 1. Associations of adiposity measures (per standard deviation change) with measures of aortic stiffness in participants without cardiovascular disease (N=1183).

Adiposity variable	niCFPWV	FWA	Z_C
	β±SE (*P*)	*β*±SE (*P*)	*β*±SE (*P*)
Waist-to-height ratio	-0.04±0.03 (0.11)	0.00±0.02 (0.88)	-0.07±0.03 (0.02)
Body mass index	-0.09±0.03 (<0.001)	0.01±0.02 (0.74)	-0.07±0.03 (0.02)
VAT*	0.14±0.03 (<0.001)	0.08±0.03 (0.005)	0.05±0.03 (0.10)
SAT*	-0.07±0.05 (0.16)	0.00±0.05 (0.98)	0.05±0.05 (0.38)

niCFPWV, negative inverse carotid-femoral pulse wave velocity. FWA, forward wave amplitude. Z_c, characteristic impedance. VAT, abdominal visceral adipose tissue. SAT, abdominal subcutaneous adipose tissue. Regression estimates (*β*) followed by the standard error (SE) and *P* values. All coefficients represent SD difference in adiposity variables per SD difference in hemodynamic variables. All models are additionally adjusted for age, age^2, sex, heart rate, mean arterial pressure, diabetes, use of antihypertensive medication, use of lipid-lowering medication, fasting glucose, total/high-density lipoprotein cholesterol ratio, and smoking status. *Models additionally adjusted for body mass index and time between computed tomography and tonometry assessment. Bonferroni-adjusted *P* values (*P*=0.05/12=0.0042) were used to assess significance of associations.

Supplemental Table 2. Associations of adiposity measures (per standard deviation change) with measures of microvascular function in participants without cardiovascular disease (N=1534).

Adiposity variable	Baseline flow velocity<br>*β*±SE (*P*)	Hyperemic flow velocity<br>*β*±SE (*P*)
Waist-to-height ratio	0.08±0.03 (0.003)	0.03±0.03 (0.22)
Body mass index	0.09±0.03 (<0.001)	0.04±0.03 (0.10)
VAT*	0.06±0.03 (0.08)	0.00±0.03 (0.89)
SAT*	-0.04±0.05 (0.41)	-0.05±0.05 (0.25)

Regression estimates (*β*) followed by the standard error (SE) and *P* values. All coefficients represent SD difference in adiposity variables per SD difference in flow velocity variables. All models are additionally adjusted for age, age^2, sex, heart rate, mean arterial pressure, diabetes, use of antihypertensive medication, use of lipid-lowering medication, fasting glucose, total/high-density lipoprotein cholesterol ratio, and smoking status. *Models additionally adjusted for body mass index and time between computed tomography and ultrasound assessment. VAT, abdominal visceral adipose tissue. SAT, abdominal subcutaneous adipose tissue. Bonferroni-adjusted *P* values (*P*=0.05/8=0.006) were used to assess significance of associations.

Supplemental Table 3. Summary of interactions for relations of aortic stiffness with body mass index and abdominal visceral adipose tissue.

Variable	niCFPWV *P*	FWA *P*	Z_c *P*
Body mass index			
Interaction by below vs. at/above median age	**0.01**	---	0.76
Interaction by sex	0.23	---	0.26
Interaction by presence of diabetes	0.08	---	0.08
Interaction by presence of metabolic syndrome*	0.52	---	0.90
Abdominal visceral adipose tissue			
Interaction by below vs. at/above median age†	**0.005**	0.19	---
Interaction by sex†	0.54	0.29	---
Interaction by presence of diabetes†	0.78	0.69	---
Interaction by presence of metabolic syndrome*†	0.16	0.77	---

niCFPWV, negative inverse carotid-femoral pulse wave velocity. FWV, forward wave amplitude. Zc, characteristic impedance. *P*-values for interaction terms are presented. All models are adjusted for age, age^2, sex, heart rate, mean arterial pressure, diabetes, prevalent cardiovascular disease, use of antihypertensive medication, use of lipid-lowering medication, fasting glucose, total/high-density lipoprotein cholesterol ratio, and smoking status. *Models additionally adjusted for presence of metabolic syndrome. †Models additionally adjusted for body mass index and time between computed tomography and tonometry assessment. We did not observe significant associations of aortic stiffness measures with waist-to-height ratio and subcutaneous adipose tissue.

Supplemental Table 4. Summary of interactions for relations of baseline brachial flow velocity with waist-to-height ratio and body mass index.

Variable	*P*
Waist-to-height ratio	
Interaction by below vs. at/above median age	0.53
Interaction by sex	0.32
Interaction by presence of diabetes	0.34
Interaction by presence of metabolic syndrome*	**0.01**
Interaction by below vs. at/above median CFPWV	0.29
Body mass index	
Interaction by below vs. at/above median age	0.32
Interaction by sex	0.16
Interaction by presence of diabetes	0.78
Interaction by presence of metabolic syndrome*	**0.04**
Interaction by below vs. at/above median CFPWV	0.06

P-values for interaction terms are presented. All models are adjusted for age, age^2, sex, heart rate, mean arterial pressure, diabetes, prevalent cardiovascular disease, use of antihypertensive medication, use of lipid-lowering medication, fasting glucose, total/high-density lipoprotein cholesterol ratio, and smoking status. *Models additionally adjusted for presence of metabolic syndrome. We did not observe significant associations of aortic stiffness measures with waist-to-height ratio and subcutaneous adipose tissue. We did not observe significant associations of baseline brachial flow velocity with abdominal visceral and subcutaneous adipose tissue.
